# Children With Dyslexia and Typical Readers: Sex-Based Choline Differences Revealed Using Proton Magnetic Resonance Spectroscopy Acquired Within Anterior Cingulate Cortex

**DOI:** 10.3389/fnhum.2018.00466

**Published:** 2018-11-23

**Authors:** Tzipi Horowitz-Kraus, Kelly J. Brunst, Kim M. Cecil

**Affiliations:** ^1^Educational Neuroimaging Center, Faculty of Biomedical Engineering – Faculty of Education in Science and Technology, Technicon – Israel Institute of Technology, Haifa, Israel; ^2^Reading and Literacy Discovery Center, Department of Pediatrics, University of Cincinnati College of Medicine, Cincinnati, OH, United States; ^3^Division of General and Community Pediatrics, Department of Pediatrics, University of Cincinnati College of Medicine, Cincinnati, OH, United States; ^4^Department of Environmental Health, University of Cincinnati College of Medicine, Cincinnati, OH, United States; ^5^Imaging Research Center, Cincinnati Children’s Hospital Medical Center, Cincinnati, OH, United States; ^6^Department of Radiology, University of Cincinnati College of Medicine, Cincinnati, OH, United States

**Keywords:** dyslexia, MRI, spectroscopy, reading, executive functions

## Abstract

Children with dyslexia exhibit slow and inaccurate reading, as well as problems in executive functions. Decreased signal activation in brain regions related to visual processing and executive functions has been observed with functional magnetic resonance imaging with reports of sex differences in brain patterns for visual processing regions. However, the underlying neurochemistry associated with deficits in executive functions for children with dyslexia has not been thoroughly evaluated. Reading ability and executive functions were assessed in fifty-three children [ages 8–12 years old, dyslexia (*n* = 24), and typical readers (*n* = 30)]. We employed short echo, single voxel, proton magnetic resonance spectroscopy to evaluate the perigenual anterior cingulate cortex (ACC). Pearson correlations were calculated between metabolite concentrations and measures of reading, processing speed, and executive function. Logistic regression models were used to determine the effects of brain metabolite concentrations, processing speed, and reading scores on dyslexia status. Differences by child’s sex were also examined. Compared to typical readers, higher global executive composite t-score is associated with greater odds for dyslexia (OR 1.14; 95% CI 1.05, 1.23); increased processing speed appears to be protective for dyslexia (OR 0.95; 95% 0.89–1.00). After adjustment for multiple comparisons, females with dyslexia showed strong and significant negative correlations between processing speed and myo-inositol (*r* = -0.55, *p* = 0.005) and choline (*r* = -0.54, *p* = 0.005) concentrations; effect modification by sex was confirmed in linear regression models (p_sex∗Cho_ = 0.0006) and (p_sex∗mI_ = 0.01). These associations were not observed for males or the group as a whole. These findings suggest that children with dyslexia share difficulty in one or more areas of executive function, specifically those related to response time. Also, metabolite changes in the ACC may be present in children with dyslexia, especially for females, and may hold value as possible markers for dyslexia.

## Highlights

- Compared to typical readers, higher global executive composite t-score is associated with greater odds for dyslexia.
- Increased processing speed appears to be protective for dyslexia.
- Processing speed in females was negatively correlated with perigenual anterior cingulate concentrations of choline and myo-inositol.

## Introduction

### Reading Difficulties and Executive Functions

Reading difficulties (or dyslexia) are characterized by slow and inaccurate reading which continues into adulthood despite remedial intervention and exposure to the written language (International Dyslexia Association IDA, 2011). Specific challenges related to phonological and orthographical processing deficits, and more broadly the reading process, have been observed in individuals with dyslexia (Seidenberg and McClelland, 1989; Pugh et al., 2000). However, our accumulated data suggests that children with reading difficulties also demonstrate challenges in several higher order abilities, i.e., in executive functions (Horowitz-Kraus, 2014). More specifically, we found that children and adults with dyslexia demonstrate decreased error monitoring ability, as manifested using electroencephalographic (EEG)-event related potentials (ERP) amplitudes following a commission of an error (i.e., decreased error related negativity potential) compared to age matched typical readers (Horowitz-Kraus and Breznitz, 2008, 2013; Horowitz-Kraus, 2011, 2012). This alteration was not specific to reading materials but was also extended to tasks which do not contain verbal stimulation (i.e., the Wisconsin task) (Horowitz-Kraus, 2014). This ERP pattern, which is related to a mismatch between an actual and a desired response, is an evoked post-response and is related to a change in dopaminergic serge (Falkenstein et al., 1991, 2000). The error related negativity evoked from the anterior cingulate cortex (ACC) (Falkenstein et al., 2000) is part of the error detection system in the brain. The ACC is a critical part of the cingulo-opercular network which is related to a top-down monitoring process (Dosenbach et al., 2008). Support for the altered brain activation related to the ACC was found in our study showing a decreased functional connection within the cingulo-opercular network during reading in children with dyslexia and an increased functional connectivity following intervention that accompanied reading achievement (Horowitz-Kraus et al., 2015b). Since decreased ERPs are related to decreased neuronal firing, which in turn points at decreased signal activation on functional magnetic resonance imaging (fMRI), it is therefore not surprising that this network showed decreased functional connections with visual processing during a reading task in children with dyslexia, and altered functional connection during a Stroop task in this population (Levinson et al., 2018) emphasizing its critical role in monitoring during reading (Horowitz-Kraus et al., 2015a) as well as in the absence of a task (i.e., during rest) (Horowitz-Kraus et al., 2015b). Other ERP and fMRI studies revealed differences between individuals with dyslexia and typical readers, including manipulations aimed at improving reading through a specific triggering of executive functions, increased ERPs related to error monitoring (Horowitz-Kraus and Breznitz, 2014), and increased activation of the ACC (Horowitz-Kraus et al., 2014) and of functional connectivity of the cingulo-opercular network (Horowitz-Kraus et al., 2015b). These EEG and fMRI findings led us to examine the specific differences in metabolite concentrations within the ACC mediating reading ability.

### Are There Neurochemical Characteristics for Reading Difficulties?

Lebel et al. (2016) observed using proton magnetic resonance spectroscopy (MRS) in typical preschool children that phonological processing ability was positively associated with glutamate (Glu), creatine (Cr), and myo-inositol (mI) concentrations in the pregenual anterior cingulate. As mI concentrations are thought to have a role as a marker of glial cells, the increased levels supported increased Glu neurotransmission and this metabolism provided a coupled relationship with phonological processing. However, most studies in the field of dyslexia focus on the role of phonological and orthographical routes (Shaywitz and Shaywitz, 2003a,b; Shaywitz et al., 2003). Pugh et al. (2014) characterized metabolite levels in children with dyslexia within the occipital cortex. This study employed English speaking, 6–10 year old typical readers and individuals with dyslexia and suggested a negative correlation between Cho and Glu concentrations with reading and phonological processing abilities (Pugh et al., 2014). The authors speculated that elevated Glu, a marker for hyperexcitability, may influence the coherence of neuronal networks involved in learning, which may also be the case for other pathologies. These researchers suggested that an unstable performance of children with reading difficulties is characterized with a moment to moment variance in performance and a lack of consistency (Pugh et al., 2014). This may provide a link to the monitoring challenges found in this population, which are not restricted to the reading domain. In a subsequent study, Del Tufo et al. (2018) found in children that cross-modal word matching mediates the relationship with increased Glu and increased Cho with poorer reading ability. Given that Cho levels represent membrane turnover as well as cellular and white matter density (Miller et al., 1996), elevated levels are in-turn related to increased connectivity or atypical myelination of the occipital cortex with additional regions in children with dyslexia (Pugh et al., 2014). Similarly, higher Cho levels in the angular gyrus are also associated with low reading scores in adults (Bruno et al., 2013). There it was suggested that Cho may be related to less efficient neuronal activity in this regions related to phonological processing and lower reading scores (Bruno et al., 2013). Bruno et al. (2013) also indicated that N-acetylaspartate (NAA) levels in the angular gyrus, a marker for intact neuronal ability, was related to a higher cognitive ability in adults (Jung et al., 2005). Del Tufo et al. (2018) also found that higher NAA predicted faster cross-modal matching reaction times in children. Increased NAA in the prefrontal cortex was also related to enhanced metabolic rates in these regions, probably due to differences in prefrontal maturation, as was observed in Asperger patients (Murphy et al., 2002). Higher NAA was previously related as a marker of viable neurons (Erickson et al., 2012) and there is conflicting evidence of positive and negative correlations (Mohamed et al., 2010) of NAA levels and cognitive control abilities. However, there is still a gap in knowledge as to the significance of metabolite concentrations in regions specifically related to executive functions in children with dyslexia.

Therefore, the goals of the current study were to: (1) determine whether metabolite concentrations in the ACC predict silent reading and sight word efficiency, processing speed or executive functions, (2) evaluate whether metabolite concentrations, reading ability, processing speed or executive functions vary in children with dyslexia compared to typical readers, and (3) determine if the associations vary by sex.

## Materials and Methods

### Participants

Participants were recruited from posted ads and through commercial advertisements. All participants gave their informed written assent and their parents provided informed written consents prior to inclusion in the study. This study was approved by the Cincinnati Children’s Hospital Medical Center Institutional Review Board.

### Behavioral Measures

#### Baseline Reading Measures Used to Confirm Dyslexia Status

We confirmed the existence of reading difficulties using a battery of normative reading tests in English. Children with dyslexia were all diagnosed as having difficulty reading. Inclusion criteria for the dyslexia group were standard score of -1 and below or meeting the 25% or below cutoff in words reading, decoding and comprehension abilities. This battery included (a) words reading accuracy/orthography: the “Letter-Word” subset from the Woodcock and Johnson-III (WJ-III) battery (Woodcock and Johnson, 1989); (b) decoding: the “Word Attack” subset from the WJ-III (Woodcock and Johnson, 1989); and (c) reading comprehension subset from the WJ-III (Horowitz-Kraus et al., 2016). Participants in the typical readers group were age-matched students who volunteered for the study with fluent and accurate reading according to established normative levels for the WJ-III.

#### Reading Abilities

We further evaluated children’s reading abilities using the Tests of Word Reading Efficiency (TOWRE) to assess the participant’s ability to pronounce printed words (sight word efficiency) and the Tests of Silent Reading Efficiency and Comprehension (TORSEC) to assess the participant’s fluency ability.

#### Executive Functions and Cognitive Abilities

To assess executive functions and cognitive abilities, we used the Behavioral Rating Inventory of Executive Functions (BRIEF) (Gioia et al., 2002) parents questionnaire as well as the speed of processing tests from the Wechsler Intelligence Scale for Children, Fifth Edition (WISC-V) (Wechsler, 2014). The BRIEF questionnaire includes questions covering the child’s cognitive abilities (i.e., inhibition, organization, attention, monitoring, and emotional regulation). In the speed of processing subtests of the WISC-V, participants performed the symbol search and the coding sub-tests, both result in the Processing Speed Index (PSI) employed for the analyses.

### Magnetic Resonance Measures

#### Acquisition Methods

**FIGURE 1:**
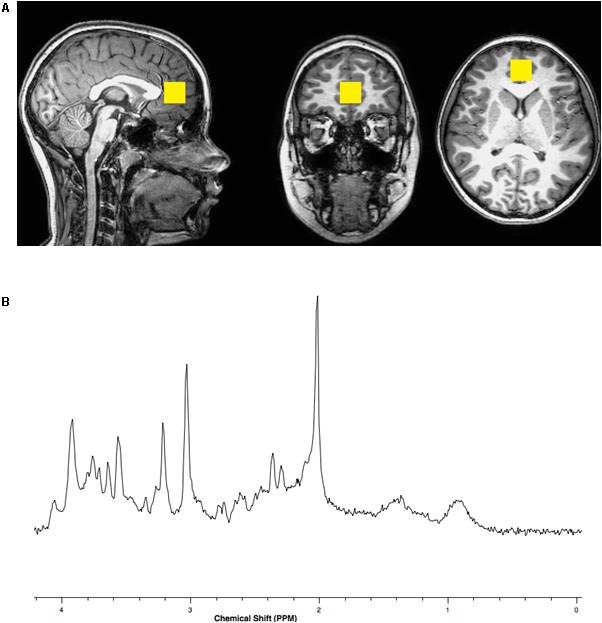
**(A)** Representative location within the anterior cingulate cortex for the 8 cubic centimeter (2 cm per side) spectroscopic voxel positioned on T1 weighted imaging slices centered in the sagittal, coronal and axial plane orientations. **(B)** Representative single voxel, short echo, magnetic resonance spectrum with N-acetylasparate appearing on the *x*-axis with a peak at a chemical shift value of 2 parts per million (PPM), glutamate at 2.3–2.4 PPM, creatine at 3.0 PPM, choline at 3.2 PPM and myo-inositol at 3.5 PPM.

Brain magnetic resonance imaging (MRI) and spectroscopy (MRS) were acquired using a Philips Achieva MR scanner operating at 3 Tesla (3T) and equipped with a 32-channel head coil. A three-dimensional (3D), high-resolution, isotropic, T1-weighted fast Fourier echo (FFE) anatomical imaging sequence was performed using 8.2 ms repetition time (TR), 3.7 ms echo time (TE), 1057 ms inversion time (TI), 8 degree flip angle, sensitivity encoding factor (SENSE) of 2, contiguous slices with a 1 mm thickness, and 1 × 1 mm voxel size. A single voxel, point resolved spectroscopy (PRESS) sequence was conducted using a 2000 ms TR, 30 ms TE, and 96 averages with water suppression along with an embedded unsuppressed water reference series of 16 averages. The 8 cubic centimeter single voxel was prescribed about the perigenual ACC within the medial frontal lobe localized from the 3D T1-FFE anatomical imaging sequence similar to the position described by Lebel et al. (2016) See Figure 1.

Participants were acclimated and desensitized to condition them for comfort inside the scanner (see Byars et al., 2002 for details). Head motions were controlled using elastic straps that were attached to either side of the head-coil apparatus. An MRI-compatible audio/visual system (Avotec, SS3150/ SS7100) was used for the presentation of a movie during the session.

#### MRS Data Analysis

The raw spectroscopy data were imported into LCModel (Provencher, 1993) commercial software for quantitative processing. Metabolite and water reference levels were determined in institutional units. The raw metabolite levels were adjusted for the tissue contributions from gray matter, white matter and cerebrospinal fluid (CSF) using FSL (Woolrich et al., 2009) adjusted to the T1 and T2 relaxation decay rate of the corrected water concentration and corrected for literature reported T1 and T2 relaxation decay rates of the primary metabolites including NAA, Cho, Cr, and mI (Wansapura et al., 1999; Traber et al., 2004; Tal et al., 2012). However, pure Glu as well as the combined glutamate and glutamine (GLX) concentrations were unadjusted for metabolite T1 and T2 relaxation decay (Gussew et al., 2012). Reported values were concentrations in units of millimolar (mM).

### Statistical Analyses

First, two sample *t*-tests were used to examine differences in age and IQ measures, reading and executive function measures as well as metabolite concentrations among children with dyslexia and typical readers.

Second, Pearson (*r*) correlations were calculated between individual metabolite concentrations and reading scales, processing speed, and global executive function. Correlation differences by dyslexia status and sex were examined. Correction for multiple comparisons was conducted by False Discovery Rate (FDR) estimation.

Third, significant correlations (FDR cutoff of 0.05) were then evaluated by linear regression adjusting for child’s sex and age at visit/test. Effect modification by sex was investigated by (1) including a two-way interaction term between metabolite concentration and child’s sex in the model, and (2) performing sex-stratified linear regression if the two-way interaction term was significant (*p* < 0.05). Effect sizes (β) represent the change in outcome for an interquartile range (IQR) increase in metabolite concentration.

Fourth, to determine if metabolite concentrations, processing speed, and global executive function predict dyslexia status, logistic regression models were implemented. For logistic models of metabolites, all metabolites were included in the same model after the investigation of multi-collinearity (based on correlations among metabolites).

Lastly, to confirm reading difficulty among children with dyslexia, we evaluated the association between silent reading and sight word efficiency and dyslexia status using the TOWRE and TOSREC. While it is likely these measures would be associated with dyslexia, it should be noted that these specific measures were not used to define the individuals in our study (see section 2.2.1). Logistic models were adjusted for child’s sex and age at visit/testing. Effect modification was evaluated by including a two-way interaction term between reading/behavior measure and child’s sex in the model and by stratification if interaction term was significant. Analyses were conducted with SAS 9.4 (SAS Institute Inc., Cary, NC, United States) and SPSS13 (IBM, Armonk, NY, United States).

## Results

### Participants

Twenty four children with dyslexia (mean age = 9.79 years, *SD* = 1.11, 8 females) and 30 typical readers (mean age = 10.73 years, *SD* = 1.07 year; 17 females) participated in the current study, all matched for age [*t*(51) = -1.77, non-significant (ns)]. All participants were within the normal range of non-verbal IQ (mean 101.45, *SD* = 10.26; children with dyslexia, mean 98.69, *SD* = 11.81 and typical readers mean 103.57, *SD* = 8.49) with [*t*(51) = -1.75, ns].

All participants were native English speakers, Caucasian, and with United States average socioeconomic status by income and education, as reported by the families using a socio-economic status questionnaire (Barratt, 2006) Participants were right-handed, displayed normal or corrected-to-normal vision in both eyes, and had normal hearing. None had a history of neurological or mood disorders, and individuals with reported history of attention difficulties were excluded.

### Behavioral Measures

Overall, children with dyslexia demonstrated significant lower scores in all reading measures taken in the study: these readers showed lower phonological and orthographical abilities. Executive functions and speed of processing in children with dyslexia were significantly lower than typical readers. See Table 1.

**Table 1:** Group assessment results for children with dyslexia and typical readers.

Assessment	Group	Mean	Standard deviation	T (*p*)
**Baseline measures**				
Test of non-verbal intelligence (percentile)	Dyslexia	49.61	23.542	-1.1417 (ns)
	Typical readers	57.50	17.021	
Letter word, Woodcock Johnson (standard score)	Dyslexia	89.04	12.448	-9.308 (*p* < 0.001)
	Typical readers	114.87	7.660	
Passage comprehension, Woodcock Johnson (standard score)	Dyslexia	83.70	12.893	-7.606 (*p* < 0.001)
	Typical readers	105.70	5.855	
Word attack, Woodcock Johnson (standard score)	Dyslexia	93.43	8.649	-7.007 (*p* < 0.001)
	Typical readers	109.77	8.224	
**Executive functions and reading measures**				
Speed of processing PSI, WISC (standard score)	Dyslexia	99.17	13.134	-2.431 (*p* < 0.05)
	Typical readers	107.47	11.643	
General executive functions, BRIEF (T score)	Dyslexia	54.68	9.317	4.038 (*p* < 0.001)
	Typical readers	44.97	7.989	
Fluency, TOSREC (standard score)	Dyslexia	84.27	9.171	-5.626 (*p* < 0.001)
	Typical readers	108.07	18.181	
Word reading, TOWRE, SWE (scaled score)	Dyslexia	81.91	12.210	-7.981 (*p* < 0.001)
	Typical readers	108.33	11.740	
Pseudoword reading, TOWRE, SWE (scaled score)	Dyslexia	81.13	10.476	-10.325 (*p* < 0.001)
	Typical readers	109.60	9.529	
**Metabolite concentrations**				
Myo-inositol (mM)	Dyslexia	5.76	0.43	0.686 (ns)
	Typical readers	5.66	0.58	
N-acetyl aspartate (mM)	Dyslexia	9.43	0.68	1.294 (ns)
	Typical readers	9.12	0.97	
Creatine (mM)	Dyslexia	8.08	0.39	0.630 (ns)
	Typical readers	7.99	0.55	
Choline (mM)	Dyslexia	1.63	0.11	-0.929 (ns)
	Typical readers	1.66	0.15	
Glutamate (mM)	Dyslexia	8.19	0.51	-0.355 (ns)
	Typical readers	8.24	0.59	
Glutamate and glutamine (mM)	Dyslexia	11.12	0.68	-1.32 (ns)
	Typical readers	11.41	0.84	

*Non-significant (ns), millimolar (mM), Behavioral Rating Inventory of Executive Functions (BRIEF), Global executive composite (BRIEF), Processing speed index (PSI), Tests of Word Reading Efficiency (TOWRE), Tests of Silent Reading Efficiency and Comprehension (TORSEC), sight word efficiency (TOWRE-SWE)*.

### Neurochemical-Behavior Correlations

Pearson correlation coefficient (*r*) values between mI, NAA, Cr, Cho, Glu, GLX, reading scales, processing speed, and global executive function were evaluated. There were no significant correlations observed among the group as a whole; however, there were differences observed by dyslexia status and sex (Table 2). Among children with dyslexia, there was a strong negative correlation between Cho and silent reading score (*r* = 0.51, *p* = 0.01); typical readers showed a strong negative correlation between mI and processing speed (*r* = -0.42, *p* = 0.02). After correction for multiple comparisons, no significant differences were found between group metabolite concentrations in children with dyslexia and typical readers.

**Table 2:** Pearson correlation among metabolites and reading scales, processing speed, and global executive function by sex.

Metabolite(s)	Silent reading		Sight word efficiency		Processing speed index		Global executive composite	
	Female	Male	Female	Male	Female	Male	Female	Male
*mI*	0.26	-0.20	-0.36	-0.004	-**0.55**	0.18	0.04	**0.37**
*NAA*	-0.10	-0.05	-0.15	0.12	-0.30	0.08	0.01	0.32
*Cr*	-0.29	-0.09	-0.35	0.03	-**0.49**	0.24	-0.14	0.35
*Cho*	-0.24	0.24	-0.31	0.17	-**0.54**	0.35	-0.01	-0.18
*Glu*	-0.05	-0.20	-0.23	0.003	-0.34	0.06	-0.21	-0.10
*Glx*	0.21	-0.09	-0.08	0.11	-0.16	0.26	-0.28	-0.06

*mI (myo-inositol), NAA (N-acetyl aspartate), Cr (creatine), Cho (Choline), Glu (glutamate), GLX (glutamate and glutamine), Silent reading (TOSREC), sight word efficiency (TOWRE-SWE), processing speed index (WISC), global executive composite (BRIEF). **Bold text** denotes significant correlation at *p* < 0.05. Correlations significant after correction for multiple comparisons are Cho/PSI (FDR = 0.05) and mI/PSI (FDR = 0.05). Cho and silent reading were correlated among children with dyslexia (*r* = -0.51, *p* = 0.01) and mI and processing speed were negatively correlated among typical readers (*r* = -0.42, *p* = 0.02); these were not significant after multiple comparisons*.

Females showed strong and significant negative correlations between processing speed and mI (*r* = -0.55, *p* = 0.005), Cr (*r* = -0.49, *p* = 0.01), Cho (*r* = -0.54, *p* = 0.005) levels. See Table 2. After correction for multiple comparisons, the correlations between PSI, mI, and Cho remained significant (FDRs = 0.05). Among males, there was a positive correlation between global executive function and mI (*r* = 0.35, *p* = 0.05); this did not hold up after correction for multiple comparisons (FDR = 0.49).

### Effect of Neurochemical Levels on Processing Speed

**FIGURE 2:**
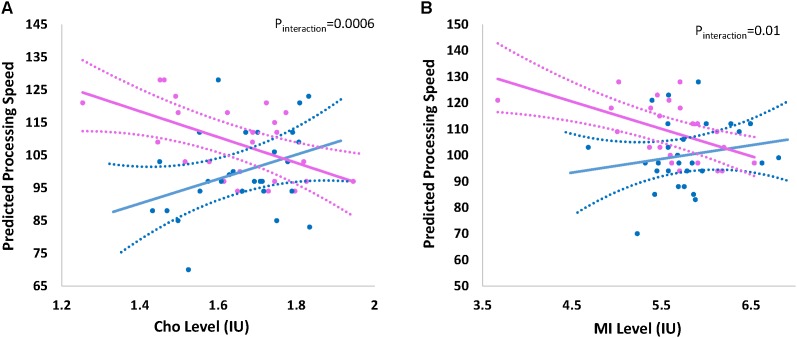
Effect of **(A)** choline and **(B)** myo-inositol on processing speed by sex. Graph represents the effect of **(A)** choline (Cho) and **(B)** myo-inositol (mI) on the predicted processing speed of participants by child’s sex Sex-stratified regression models are adjusted for age at visit/testing. Blue and pink indicates the effect for males and females, respectively. Dashed lines represent 95% confidence intervals. Actual individual level data are overlaid for males (blue circles) and females (pink circles). Interaction *p*-value represents the *p*-value for the formal two-way interaction between metabolite level and child’s sex in the linear regression model.

The overall effect of Cho and mI on processing speed was not significant; however there was indication of effect modification by child’s sex. Figure 2 shows the effect of (a) Cho (p_interaction_ = 0.0006) and (b) mI (p_interaction_ = 0.01) on predicted mean scores for processing speed by child’s sex. Higher levels of Cho (β -8.10; 95% CI -12.73, -3.45) and mI (β -5.22; 95%CI -8.22, -2.22) are associated with decreased processing speed among females but not males (Table 3).

**Table 3:** Overall and sex-stratified results for associations between choline, myo-inositol and PSI.

Model	β	95%CI	*P*-value
Overall			
Cho	-1.56	-6.15, 3.03	0.51
mI	-2.03	-5.02, 0.96	0.18
Females			
Cho	-8.10	-12.73, -3.45	**0.0006**
mI	-5.22	-8.22, -2.22	**0.0006**
Males			
Cho	7.11	-0.03, 14.25	0.06
mI	2.51	-2.49, 7.51	0.33

*Choline (Cho), myo-Inositol (mI). Overall models are adjusted for sex and child’s age at visit; sex-stratified models are adjusted for child’s age at visit/testing. β represent the change in PSI for an interquartile range (IQR) increase in metabolite (Cho IQR = 0.19 IU, MI IQR = 0.47 IU)*.

### Effect of Global Executive Function, Processing Speed, Reading Measures, and Metabolites on Dyslexia Status

Children with a higher global executive composite t-score are more likely to have dyslexia (OR 1.14; 95% CI 1.05, 1.23) suggesting some difficulty in one or more areas of executive function is present among children with dyslexia compared to typical readers (Table 4). We also observed a marginally significant (*p* = 0.049) association between increased processing speed and protective odds for dyslexia (OR 0.95; 95% 0.89–1.00) compared to typical readers. Metabolite concentrations did not predict dyslexia status. Metabolite concentrations were moderately to significantly correlated *(r* ranging from 0.22 to 0.59, *r* = 0.80 for GLX/Glu correlations) and given the strong correlation between GLX and Glu, a sensitivity analysis was conducted to determine if removal of one of these metabolites impacted the results; the findings remained insignificant. As expected, higher scores on silent reading and sight word efficiency are observed among typical readers as evident by the ORs less than 1 (Table 4). There was no evidence of effect modification by child’s sex (all interaction *p*-values > 0.20).

**Table 4:** Relationship between reading scales, processing speed, global executive function, and metabolite levels on dyslexia status.

Predictor(s)	OR	95%CI	*P*-value
Silent reading	0.88	0.82, 0.95	0.001
Sight word efficiency	0.85	0.77, 0.92	0.0002
Processing speed	0.95	0.89, 1.00	0.049
Global executive composite	1.14	1.05, 1.23	0.002
Metabolite predictors^a^			
mI	1.439	0.193, 10.72	0.72
NAA	1.563	0.530, 4.62	0.41
Cr	4.078	0.459, 36.20	0.21
Cho	0.006	0.001, 4.48	0.13
GLX	0.353	0.069, 1.80	0.21
Glu	1.368	0.137, 13.65	0.79

^a^*Metabolites are included in the same model; All models are adjusted for sex and child’s age at visit and represent the odds of having dyslexia. Behavioral Rating Inventory of Executive Functions (BRIEF), Tests of Word Reading Efficiency (TOWRE), Tests of Silent Reading Efficiency and Comprehension (TORSEC). Silent reading (TOSREC-index), sight word efficiency (TOWRE–SWE), processing speed index (WISC–PSI), global executive composite (BRIEF–GEC T score). mI (myo-inositol), NAA (N-acetyl aspartate), Cr (creatine), Cho (Choline), Glu (glutamate), GLX (glutamate and glutamine)*.

## Discussion

Over the past decade, multiple studies have implicated difficulties in executive functions for individuals with dyslexia. However, there is still a gap in knowledge as to the neurochemical characteristics related to reading difficulties in neural circuits associated with challenges in executive functions for this population. The current study aimed to characterize the metabolite concentrations related to reading ability in typical readers and in children with dyslexia, specifically focusing on the ACC. As hypothesized, lower reading ability was associated with high Cho levels, however, it was negatively associated with processing speed in females with dyslexia. The current study also demonstrated a negative relationship between processing speed and mI concentration. To our knowledge, this is among the first pediatric studies to look at MRS measures in the ACC and to report higher levels of Cho in the brain of females with dyslexia. Also, the study confirmed children, both boys and girls, with dyslexia demonstrated lower reading ability and executive functions scores compared to typical readers.

The study did not observe relationships of the cognitive and reading measures with other metabolites, such as NAA, Glu, or GLX, nor did it observe metabolite changes in males. In this study, metabolite concentrations were determined with corrections for metabolite and water relaxation along with contributions from different tissue types contributing to the voxel. Other studies report relative metabolite levels to one another, usually to creatine, and fail to account for differences in tissue contributions, which may explain observed differences. The voxel placement within the pregenual ACC in the current study matches the location described by Lebel et al. (2016) however, it differs from that of other studies of individuals with dyslexia focused on the occipital lobe including lingual gyrus, calcarine sulcus, and cuneus.

### High Cho and Reading Difficulty

Previous studies have pointed at high Cho levels related to reading levels in the occipital lobe (Pugh et al., 2014; Del Tufo et al., 2018) in the cerebellum (Laycock et al., 2008) and the left temporoparietal region (Bruno et al., 2013). The authors suggested that these high Cho levels in individuals with reading difficulties for reading-related regions reflect high membrane turnover, cellular density, and white matter density. These results are in line with evidence of impaired myelination in this population in white matter tracts passing the temporoparietal regions and the occipital lobe (Yeatman et al., 2012; Wandell and Yeatman, 2013). Our study is believed to be among the first to reveal metabolite changes in regions that were not traditionally included as part of the classical reading circuitry. However, in recent years there are additional models pointing at the critical role of executive functions-related neural circuits in reading, including the ACC (Horowitz-Kraus et al., 2013, 2018; Horowitz-Kraus and Hutton, 2015). Our results provide additional evidence for the involvement of neural circuits related to executive functions in children with dyslexia extending the high Cho findings also to the ACC. The relationship to alterations in myelination (especially in the genu which passes though the ACC) should also be examined. As the ACC in a major hub in the cingulo-opercular network, (Dosenbach et al., 2008) it would be interesting to measure Cho levels in other sub-regions of the ACC (as outlined in) (de la Vega et al., 2016) and other brain regions that are related to the fronto-parietal network and the fast monitoring of cognitive processes also impaired in children with dyslexia.

### Differences in mI Between Children With Dyslexia and Typical Readers

myo-inositol is a carbocyclic sugar molecule localized to glial cells. There are several known roles for mI in the brain. It is primarily considered a glial cell marker as it’s concentration increases with glial cell based neoplasms and gliosis. However, it also functions as an osmolyte such that during periods of osmotic stress, as balance is maintained via mI transport across plasma membranes. However, in the context of the current study with Cho findings, there is support for the role of mI as a key precursor of phosphoinositides and phospholipids, cell membranes and myelin structures. The composition of these structures within the brain networks could directly influence reading abilities.

### Sex-Specific Findings

We were able to explore sex-based differences as eight of twenty-four children with dyslexia and seventeen of thirty typical readers in our study were females. Sex differences are known in many disorders, including ADHD, and dyslexia, with greater male frequency with the diagnosis (Shaywitz and Shaywitz, 2003a,b, 2008). Multiple genetic, and non-inherited factors have been posited to explain this observation. The sex hormones, estrogen and progesterone, demonstrate cognitive and neuroprotective effects (Brann et al., 2007; Dumitriu et al., 2010).

Arnett et al. (2017) concluded that the higher prevalence in males with reading difficulties can be explained by slower and more variable processing speed along with worse inhibitory control. Geschwind and Levitsky (1968) first reported structural differences and asymmetry in persons with dyslexia upon post-mortem brain examinations. Non-invasive neuroimaging investigations of brain structure have provided further insight into the neurobiological basis for sex-specific differences. Evans et al., found gray matter volume differences in adults and children, male and female with developmental dyslexia, specifically in the occipital lobe for girls (Evans et al., 2014).

### Study’s Limitations

The results of this study should be considered in light of the following limitations. First, the sample size of this study is relatively small. Second, the perigenual placement of the MRS voxel within the ACC limited the ability to relate to executive functions of the ACC assigned to more posterior aspects, such as cognitive control and conflict monitoring. Third, technical differences in the MRS acquisition and quantitation of metabolite levels, as previously discussed, can influence the results. Also, the relationship between BOLD and functional connectivity and our findings should be validated in a future study using a joint MRS-fMRI model combining the results of these two methodologies.

## Conclusion

The current study’s findings pinpoint metabolic differences related to the medial frontal lobe in females with dyslexia and typical readers. Further investigations are necessary to explore the metabolism and function of the ACC and how they influence reading abilities.

## Author Contributions

All authors contributed to data analysis and manuscript writing.

## Conflict of Interest Statement

The authors declare that the research was conducted in the absence of any commercial or financial relationships that could be construed as a potential conflict of interest.
